# Duration of shedding of respiratory syncytial virus in a community study of Kenyan children

**DOI:** 10.1186/1471-2334-10-15

**Published:** 2010-01-22

**Authors:** Emelda A Okiro, Lisa J White, Mwanajuma Ngama, Patricia A Cane, Graham F Medley, D James Nokes

**Affiliations:** 1 Malaria Public Health & Epidemiology Group, Centre for Geographic Medicine Research - Coast, Kenya Medical Research Institute, Nairobi, Kenya; 2 Centre for Tropical Medicine, University of Oxford, John Radcliffe Hospital, Headington, Oxford, UK; 3 Centre for Clinical Vaccinology and Tropical Medicine, Nuffield Department of Clinical Medicine, John Radcliffe Hospital, University of Oxford, Oxford, UK; 4 Faculty of Tropical Medicine, Mahidol University, Bangkok, Thailand; 5 Centre for Geographic Medicine Research - Coast, Kenya Medical Research Institute, Kilifi, Kenya; 6 Centre for Infection, Health Protection Agency, London, UK; 7 Department of Biological Sciences, University of Warwick, Coventry, UK

## Abstract

**Background:**

Our understanding of the transmission dynamics of respiratory syncytial virus (RSV) infection will be better informed with improved data on the patterns of shedding in cases not limited only to hospital admissions.

**Methods:**

In a household study, children testing RSV positive by direct immunofluorescent antibody test (DFA) were enrolled. Nasal washings were scheduled right away, then every three days until day 14, every 7 days until day 28 and every 2 weeks until a maximum of 16 weeks, or until the first DFA negative RSV specimen. The relationship between host factors, illness severity and viral shedding was investigated using Cox regression methods.

**Results:**

From 151 families a total of 193 children were enrolled with a median age of 21 months (range 1-164 months), 10% infants and 46% male. The rate of recovery from infection was 0.22/person/day (95% CI 0.19-0.25) equivalent to a mean duration of shedding of 4.5 days (95%CI 4.0-5.3), with a median duration of shedding of 4 days (IQR 2-6, range 1-14). Children with a history of RSV infection had a 40% increased rate of recovery i.e. shorter duration of viral shedding (hazard ratio 1.4, 95% CI 1.01-1.86). The rate of cessation of shedding did not differ significantly between males and females, by severity of infection or by age.

**Conclusion:**

We provide evidence of a relationship between the duration of shedding and history of infection, which may have a bearing on the relative role of primary versus re-infections in RSV transmission in the community.

## Background

Respiratory syncytial virus (RSV) is the major viral cause of lower respiratory tract infection in children worldwide [1-4]. Our understanding of the mechanisms of persistence and spread of the virus in the population is fundamental to the development of appropriate control methods. In this respect the process of recovery from infection is of intrinsic interest. Primary RSV infection predominantly arises in the first two years of life [5-10]. However, due to imperfect immunity RSV repeatedly infects, probably throughout life [11,12]. Given that most infections are therefore repeat infections, their role in the overall transmission of the virus within a population is potentially of fundamental importance, and highly dependent upon the level and duration of shedding [13]. Roughly 1 in 100 cases of infant primary RSV infection result in hospitalisation and a far smaller proportion in older ages and following re-infection [5,14]. Hence, measurement of the infectivity of community cases not found in the hospital setting is important; particularly if severity is related to duration of shedding as such cases are likely to be the main contributors to transmission. It is recognised that immunocompromised individuals can shed RSV for many weeks, and persistent shedding may have significant impact on RSV persistence and seasonal patterns [15].

It is therefore surprising that published estimates of the duration of shedding of RSV are few. Studies of children hospitalized with acute respiratory infection (ARI) [16-19], provide estimates of shedding duration biased towards infections causing severe disease and from a narrow (young) age group. It has been shown that greater severity of infection within in-patients results in increased duration of shedding [17]. One study estimated the duration of RSV shedding through home-based monitoring of all individuals in 36 U.S. families during a single epidemic [11]. A mean duration of 3.5-7.4 days in otherwise healthy children was recorded (range 1-36 days), with higher duration for children under 2 years than for children under 16 years (9 vs. 3.9 days). Estimates, however, were not defined in relation to past infection status. A second study of 97 U.S. families following children under 4 years of age reported over 70% of cultures positive up to 7 days post illness onset, falling to less than 10% in week 2 and 3 [20]. No evidence for a difference in shedding duration between primary and re-infections was observed in this study (there were only 16 re-infections). Studies of adults [21] in the community show shedding to be of similar or slightly shorter duration than shedding for children 1.6-3.9 days (range 1-27). Despite the fact that the median duration of RSV shedding tends to be of the order of days, considerable variation exists: in the family studies referred to earlier, 5-10% of individuals shed for more than 14 days, and periods of shedding of 30-40 days have been recorded[11,17,18,20].

In this paper we report on the duration of virus shedding from RSV infected individuals within a family cohort in a rural Kenyan community, in relation to infection history, age and severity.

## Methods

### Study population and samples

The study was undertaken in Kilifi District, a rural coastal area of Kenya, and forms part of an epidemiological investigation of RSV through the intensive surveillance of a birth cohort [4,14] and a household cohort [11,12,22]. Infants numbering 635 were recruited at birth or within two weeks of birth from January to June 2002 and from December 2002 to May 2003 and intensively monitored for acute respiratory infections (ARI) over three RSV epidemics. A sub-sample of 70 households, each with one or more siblings of birth cohort children, was enrolled into a family study. All family members were monitored for ARI for a period including two RSV epidemics (Dec 2003-June 2004 and November 2004-March 2005) through weekly household visits during epidemics and monthly otherwise, self referral to a research out-patient clinic and admission to a paediatric ward of the District hospital. Data on the onset of symptoms defined by history was recorded at presentation to the research clinic. Nasal washings (NW) using a nasal wash bulb method were collected from infants and elder household siblings (<15 years of age; range 0-14) experiencing episodes of acute (rapid onset) respiratory illness, whether mild (e.g. runny nose) or more severe. The presence or history of these symptoms in the preceding week was used as a prompt for sample collection. Respiratory disease severity was assigned as upper respiratory tract infection (URTI), mild lower respiratory tract infection (LRTI), or severe LRTI or very severe LRTI as previously described [4]. Mild LRTI was defined as a history of acute cough or difficulty in breathing and fast breathing for age. Severe (inclusive of very severe) LRTI was defined as a history of acute cough or difficulty in breathing and one of more of the following: (1) indrawing, (2) low oxygen saturation (<90%) by pulse oxymetry or (3) inability to feed inclusive of prostration or unconsciousness, the latter two conditions applying only if accompanied by a clinical diagnosis of LRTI or bronchiolitis. Samples were screened for RSV antigen by a commercial direct immunofluorescent antibody test (RSV DFA, Chemicon) [4]. Informed consent for participation in the study was obtained from each child's guardian. Ethical approval for the study was granted by the KEMRI/National Ethical Review Committee, in Kenya and Coventry Research Ethics Committee, in the UK.

Mothers of RSV positive children were asked to enrol their children into the shedding study. We estimated the sample size based on infection rates monitored from previous epidemics (assumed to be 30%) [4], with the expected result of approximately 100 primary cases and 100 re-infections from the family cohort. We estimated that this would enable the detection of a 50% difference in two rates (e.g. 1/9 days vs. 1/6 days for primary and secondary infections) with 90% power at the 5% significance level [23], even after stratification for one potential confounder (e.g. age class). Following the identification of RSV infection (day 1), a further NW was obtained as soon as possible, and thereafter scheduled for every 3 days up to day 14, thereafter after an additional 7 and 14 days, and subsequently every 2 weeks up to 16 weeks. During follow up no further samples were taken after a single sample tested antigen negative by DFA test.

### Data analysis

Data were analyzed using Stata version 8.0 (Statacorp, College Station, Texas, US). Time to recovery (end of shedding) survival analysis (Cox regression) was used for repeated measurements within the individual specific for each episode of infection. We defined the null hypothesis as an absence of association between the duration of viral shedding and infection history, age or severity. History of infection was defined as a previous positive sample for children followed from birth and was otherwise assumed for all children over 3 years (greater than 36 months) of age. Initially, an analysis of all records was carried out using the start date for viral shedding i.e. the first day of sampling as day 0. Subsequently, for data records from research clinic visits where relevant information was collected, the start date for viral shedding was considered to coincide with the start of the onset of symptoms defined by history taken at presentation to the research clinic with day 0 as the first day of symptoms. The end of shedding was denoted by the first negative test sample. Schoenfeld residuals [24] were used to test the proportionality assumption on which the method is based and the models were found not to violate the assumption. Shedding was assumed to start on the day of the first positive sample (or on day of onset of symptoms) with the end on the day of the first negative test sample. We used the likelihood ratio test to test the null hypothesis that two models are equally supported by the data.

## Results

**Figure 1:**
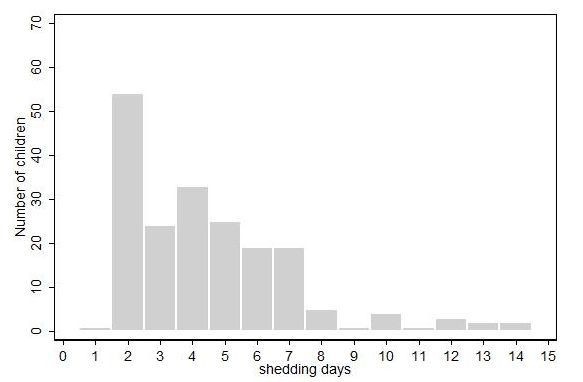
**Frequency of duration of RSV shedding**.

A total of 193 RSV positive children, from 151 families, were enrolled into the shedding study, of which 160 were birth cohort infants and 33 siblings. 192 negative results (i.e. indicating cessation of shedding) were observed. One child died in hospital before completing the study and due to lack of further information is right censored. For 120 children, the first positive sample was also the last positive sample. The children were between the ages of 2 and 164 months at recruitment into the shedding study, 10.4% were less than 1 year while 70% were 2 years of age or less. The median age of children in the study was 21 months, and 46% were male. 3% of all the children in the larger birth cohort had a birth weight of <2.5 kg. Of the 193 RSV infections 165 were classified as having an upper respiratory tract infection (URTI), 20 as mild LRTI, 8 as severe LRTI, and none with very severe LRTI. The frequency distribution of days to cessation is shown in Fig. 1. The overall rate of recovery (cessation of shedding) per day irrespective of infection history, age and severity was 0.22/person/day (95% CI 0.19-0.25) i.e. a mean duration of shedding (reciprocal of rate of recovery) of 4.5 days (95%CI 4.0-5.3), with a median duration of shedding for all children of 4 days (IQR 2-6, range 1-14). The proportion of individuals with prior history of infection was 0.53 in children with URTI compared with 0.5 and 0.25 in children with severe LRTI and mild LRTI, respectively. Both the URTI and mild LRTI disease categories had a higher proportion of children in the older (≥18 months) age group compared to the severe LRTI category. One hundred and thirty six of 193 positives were from children attending the research clinic and these records had data on the number of days with symptoms based on history. The average duration of shedding for this subset of children was 7.69 days (95% CI 6.41-8.98). A comparison of the two populations (clinic attendees and non-clinic attendees) reveals that the ages of children who attended the clinic and those seen at home were similar (t-test, P = 0.1736). All children seen at home had an URTI except for one child who had a severe LRTI and was referred to hospital for admission. By comparison 21% of children presenting to the clinic had a diagnosis of mild or severe LRTI.

### Shedding patterns in relation to gender

The rate of loss of infection did not differ significantly between males and females (Table 1 &2; hazard ratio (HR) 0.86; 95% CI 0.64-1.16).

**Table 1:** Rates of recovery from infection (cessation of shedding virus) per day estimated using survival analysis using data from 192 RSV infected Kenyan children.

Feature		Number	Mean duration of shedding	Lower CI	Upper CI
**History**	never infected	115	4.9	4.1	5.8
	infected	77	4.1	3.3	5.1
**sex**	Male	88	4.4	3.6	5.4
	Female	104	4.7	3.9	5.7
**Age group**	0-11 months	21	4.4	2.9	6.7
	12-17 months	35	4.9	3.5	6.8
	18-23 months	65	4.9	3.9	6.3
	24+ months	71	4.1	3.3	5.2
**Severity**	URTI	165	4.4	3.8	5.1
	Mild LRTI	20	5.6	3.6	8.8
	Severe LRTI	7	5.2	2.5	10.8
**Revised History^$^**	never infected	96	5.1	4.2	6.2
	infected	96	4.0	3.3	4.9

^$ ^Assumes children over 3 years (greater than 36 months old) have been previously infected.

**Table 2:** Cox regression model results to examine factors independently associated with cessation of shedding in 192 RSV infected Kenyan children.

Feature*		RR^&^	p-value	Lower CI	Upper CI
**Revised history^$^**	infected	1.37	0.04	1.01	1.86
**Sex**	Female	0.86	0.33	0.64	1.16
**Age group**	12-17 months	1.01	0.98	0.57	1.76
	18-23 months	0.91	0.73	0.54	1.53
	24+ months	1.08	0.78	0.65	1.80
**Severity**	Mild LRTI	0.69	0.15	0.42	1.14
	Severe LRTI	0.82	0.62	0.38	1.80

*baseline for each comparison is: no history of infection, male sex, 0-11 month age group and URTI severity.

^&^All estimates are adjusted for all other factors.

^$^Assumes children over 3 years (greater than 36 months old) have been previously infected.

### Shedding patterns in relation to age

**Figure 2:**
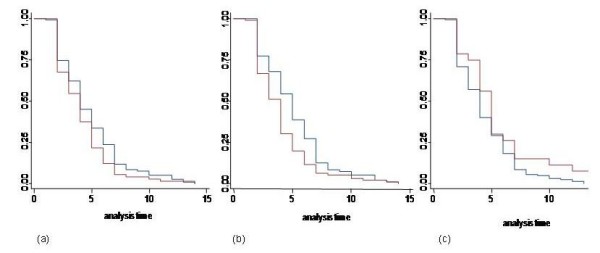
**Kaplan-Meier survivor function plots for cessation of sheddding of RSV in infected Kenyan children**. Results are categorised by age class (blue: 0-23 months; maroon: 24+ months) in Graph a, by infection history (blue: never infected; maroon: infected-significantly different; log rank test p < 0.05 ) in Graph b, and by severity (blue: URTI; maroon: LRTI) in Graph c.

Children were grouped into four age classes: 0-11 months (10%), 12-17 months (19%), 18-23 months (34%) and 24 months or more (37%). The median rate of recovery was 4 days in all the age groups with interquartile ranges of 3-5, 2-6, 3-6 and 2-5, for each age group in ascending order. The duration of shedding did not differ greatly between the four age groups as shown in Table 1. Using the 0-11 months age group as the reference group, the rate of recovery decreased by 9% (HR 0.91; 95%CI 0.54-1.53) in 18-23 months age groups, and increased by 1% (HR 1.01; 95% CI 0.57-1.76) and 8% (HR 1.08; 95%CI 0.65-1.80) in the 12-17 month and in the 24+age group, respectively. None of these differences were significant (Table 2). Reclassfiying age into two categories suggested a more rapid recovery in the older (24 months and over) relative to the younger children (Fig. 2). There was no evidence to suggest an improved fit of the model after adjusting for past history of infection (likelihood ratio test, P = 0.533).

### Shedding patterns in relation to severity

The rate of recovery was faster for children with an URTI as shown in Table 1 compared to those with mild or severe LRTI. Those with an URTI shed virus for a median of 4 days (IQR 2-6 days). Similarly, the severe LRTI cases shed virus for a median of 4 days (IQR 4-6). Those with mild LRTI shed virus for the longest period with a median of 5 days (IQR 4-6). Cox regression, suggested that relative to cases with URTI, the recovery rate decreased by 31% (HR 0.69; 95%CI 0.42-1.14) and 18% (HR 0.82; 95%CI 0.38-1.80) for those with mild and seveve LRTI, respectively (Table 2). These data are displayed graphically in Fig. 2 with the mild and severe cases combined due to small number of cases with severe LRTI. These results were not statistically significant and the model adjusting for age and history of infection did not improve the fit to the data (likelihood ratio test, P = 0.2723).

### Shedding patterns in relation to history of infection

Of the 193 children 77 had a known history of one or more past RSV infections. These were all children who had been followed up from birth. The mean duration of shedding differed between those children who had never been infected and those with prior history of infection; 4.9 (CI 4.1-5.8) days vs. 4.1 (CI 3.3-5.1) days (Table 1).

This study also included children who had been recruited as household members of a birth cohort but whose history of infection since birth was unknown. All household children who were >36 months of age (19/33 birth cohort child siblings) at the time of enrollment were classified as having had a previous infection. The duration of shedding between those children who had never been infected and those with prior history of infection after the revised assignment was 5.1 (CI 4.1-5.8) days vs. 4.0 (CI 3.3-5.1) days (Table 1). The rate of cessation of shedding was found to be significantly more rapid in individals with prior infection history relative to those without (HR is 1.37; 95%CI 1.01-1.86, P = 0.04 Table 2; Fig. 2), indicating that those with prior history had a ~40% increased rate of recovery. The median duration of shedding for the children with a previous history of infection was 4 days (median age of child 23 months) while for those without a history of infection the duration of shedding was 5 days (median age 20 months) with IQR of 2-5 and 3-7 days, respectively. Adjusting for the effect of all the other variables improved the fit to the data (likelihood ratio test, P = 0.044); this shows a significant effect of history even when controlled for age group, gender and severity (Table 2). However, these variables do not appear to confound or alter the effect of history as the estimate adjusted for these variables does not change significantly (hazard ratio of 1.41 vs 1.37).

### Shedding patterns in clinic attenders

For the subset of children presenting to the research clinic for whom illness history was obtained a separate analysis was undertaken. In this group, the duration of shedding among those children who had never been infected and those with prior history of infection (defined by antigen positive RSV detection or child age >36 months) was 8.2 (CI 6.5-10.3) days compared to 7.0 (CI 5.5-8.8) days. The rate of cessation of shedding was found to be more rapid in individuals with prior history of infection relative to those without a history of infection (Adjusted HR is 1.43; 95%CI 0.997-2.051, P = 0.052), indicative of a 43% increased rate of recovery in those children with prior history of infection.

## Discussion

Studies that investigate the duration of RSV shedding are few, especially for cases not resulting in hospital admission, and there are no studies from a resource poor setting. We followed up 193 children from 151 rural households on the coast of Kenya, estimating the mean duration of shedding to be 4.5 (95%CI 4.0-5.3) days ranging from a minimum of 1 day to a maximum of 14 days. This study provides the first estimates of shedding patterns in non-hospitalised community acquired infections in relation to past history of infection adjusted for age, sex and severity of infection.

A key finding in our study is that while the recovery from shedding was more rapid in children previously infected, the rate was only 40% higher in these children. Hence, we surmise that although primary infected children shed longer, their total contributon of days shedding virus within the community will be substantially less than the cumulative days of virus shed by children experiencing secondary infections, since due to repeated reinfections, secondary infections are far more prevalent than primary infections. A similar relationship, though more exagerated, may be inferred from the family study by Hall *et al*.[11] where longer shedding was observed for children under 2 years (who presumably had no history of infection) than for children aged 2-16 years (9 vs 3.9 days). It is of interest that the study of Frank *et al. *[20] involving approximatley 100 children found no difference in the duration of shedding between 32 primary and 16 re-infections. The study involved children within a very narrow age range (<4 years) which may explain the lack of effect seen. Methodologically that study differed from the present in that samples were collected from individuals irrespective of symptoms thus identifying shedding prior to symptoms. While this would have increased the average duration of shedding it seems unlikely to have annulled a relationship with infection history. Prolonged shedding of RSV enhances the possibility of transmission and makes such individuals potential sources of community infection an important consideration in the control and prevention of RSV infection and disease in infants [15]. In our study only two (1%) children shed virus for longer than 12 days.

No significant age-related difference in the rate of recovery was observed in this study, which accords with results reported by Wright *et al. *[25]. A plausible explanation is that the duration of shedding is determined more by immunity to infection than by a child's age (or physiology).

Evidence for a positive association between severity of the infection and duration of shedding was inconclusive in this study. Hospital based studies dealing with severe disease have observed durations of shedding of approximately 7 days or more [17,18], which is longer than the median 5 day duration of shedding for severe pneumonia in this study. We expected to see a relationship between severity and history of infection as resistance derived from past infection might be expected to reduce the severity of illness in those re-infections which do occur. Such a relationship was shown by Glezen *et al. *[5] who found that repeat infections with RSV were less severe than primary infections. However, history of infection did not appear to confound the relationship between severity and duration of shedding.

Factoring into the analysis days with symptoms for the children who presented to the clinic leads to an increased duration of shedding from approximately 5 days to 8 days. However, this makes two assumptions: First that recall of the number of days symptomatic is reliable, for which we cannot be sure. Second, that individuals begin shedding virus from the start of symptoms and that the symptoms are related to the RSV infection, which seems reasonable (Frank et al., 1981). Inclusion of days symptomatic, while increasing the estimated duration of shedding, does not alter the relationship between duration and history of past RSV infection (although statistically of borderline significance). The increased duration of shedding when prior days of symptoms are accounted for applies specifically to the more severe cases identified on our study (i.e. those serious enough to warrant clinic attendance). To what extent this increase applies also to the majority of child infections with less severe RSV who were sampled at home is not clear, though we might expect those with milder symptoms to have infections of shorter duration.

There are several methodological issues that will have resulted in the estimates reported here being minimum estimates of shedding duration. First, the duration of shedding may have been under-estimated by taking the first negative test to indicate cessation of shedding, especially as sensitivity of DFA antigen detection is not perfect and if sensitivity decreases as duration increases, due, for example, to decreasing viral load. Continued sampling for at least one more round to assess the sensitivity would have proved useful. Second, more sensitive detection techniques such as PCR might be warranted - however, it is less certain whether identification by PCR relates more to viable virus compared with the DFA diagnostic method used here; PCR has the limitation of indicating presence, but not virus viability. Third, we did not quantify the viral load in the samples. The quantification of viral load might have epidemiological consequences for transmission potential and has also been shown to be associated with disease severity [26,27]. Fourth, the data will have been subject to left censoring, i.e. exact start time of shedding was not observed. Studies that take samples irrespective of symptoms provide more information, but are methodologically difficult to implement [20]. We attempted an analysis of these data by imputing a time at infection from data on time from current infection to the last previous RSV negative DFA result, but there is very little additional information to improve the estimates from the simpler analyses reported here. Lastly, we have assumed that all children not followed up from birth who were over 36 months of age have had a prior infection, an assumption well supported by the literature [5-10]. Other approaches to detect infection history, for example examining changes in titre of specific antibodies, present their own technical problems in terms of logistics and reliability but should be considered for future studies.

## Conclusion

In summary, information on the duration of shedding of respiratory viruses in naturally infected persons that do not result in hospitalization is important to our understanding of the transmission of infection. Our findings will contribute to the development of transmission dynamic models to investigate the impact of immunization on the transmission dynamics of RSV. Specifically, we demonstrate an association between the duration of shedding and history of infection thus adding to the characterisation of the natural history of infection and helping to quantify the relative contribution of primary and secondary infections to overall infection transmission [28].

## Competing interests

The authors declare that they have no competing interests.

## Authors' contributions

EA was responsible for proposal development, study coordination, the data and the data analysis and wrote the manuscript; LJW was instrumental in the conception and design of the study, MN was the study clinician responsible for clinical care and assembling of clinical data; PAC, GFM and DJN were responsible for the project and its conception and its overall scientific management, interpretation and preparation of the final manuscript. All authors have read and approved the final manuscript.

## Pre-publication history

The pre-publication history for this paper can be accessed here:

http://www.biomedcentral.com/1471-2334/10/15/prepub
